# Quality of life instruments in idiopathic pulmonary fibrosis: a COSMIN-based systematic review

**DOI:** 10.1186/s12890-026-04550-2

**Published:** 2026-07-31

**Authors:** Enming Zhang, Jianmei Gong, Shi Wang, Yu Duan, Shuangying Ma, Haifeng Cheng

**Affiliations:** School of NursingLiaoning University of Traditional Chinese Medicine, Shenyang, Liaoning 110847 China

**Keywords:** COSMIN guideline, Idiopathic pulmonary fibrosis, Quality of life, Measurement tools, Measurement properties, Patient-reported outcome measures, Systematic review

## Abstract

**Objective:**

This systematic review aims to systematically evaluate the methodological quality and measurement properties of existing quality-of-life assessment tools for patients with idiopathic pulmonary fibrosis, and to provide evidence-based recommendations for instrument selection in clinical research and practice.

**Methods:**

A comprehensive computer search was conducted in PubMed, Embase, Web of Science, CINAHL, CNKI, Wanfang, VIP, and the Chinese Biomedical Literature Database from January 1, 2010 to December 28, 2025. Studies evaluating the measurement properties of IPF-specific quality of life tools published in Chinese or English were included. Two researchers independently screened the literature and extracted data, while four researchers assessed the methodological quality and measurement properties of the included studies according to the Consensus-Based Standards for the Selection of Health Measurement Instruments (COSMIN) guidelines.The modified Grading of Recommendations Assessment Development and Evaluation (GRADE) method was employed to grade the certainty of evidence, and based on this grading along with team consensus, recommendations were formulated.

**Results:**

A preliminary search identified 1923 records (6 Chinese, 1917 English). After removing duplicates, 910 records remained; 840 were excluded by title and abstract screening, and the remaining 70 full texts were assessed. Ultimately, 11 documents were included, encompassing 9 assessment tools. The evaluation of methodological quality revealed that most tools had methodological shortcomings, particularly in content validity, cross‑cultural validity, measurement error, and criterion validity. Following the evidence grading, none of the nine tools met the standards for Category A recommendations; all were classified as Category B recommendations. Notably, the IPF-PRO scale demonstrated higher evidence quality in terms of internal consistency, structural validity, hypothesis testing, and other measurement attributes, leading to its provisional recommendation for use.

**Conclusion:**

The IPF-PRO scale demonstrates high methodological quality in its validation study for structural validity and internal consistency, with the evidence for these properties graded as "High" by the modified GRADE approach. However, its structural validity results were rated as "indeterminate" by COSMIN criteria, indicating that the statistical findings did not fully meet the criteria for "sufficient" evidence. Content validity was graded as "Moderate" and criterion validity as "Low". Therefore, while the IPF-PRO scale shows promise and is provisionally recommended for use in Chinese clinical settings with appropriate caveats, further validation is needed to confirm its structural validity and to evaluate its measurement error and responsiveness.

**Supplementary Information:**

The online version contains supplementary material available at https://doi.org/10.1186/s12890-026-04550-2.

## Introduction

Idiopathic pulmonary fibrosis (IPF) is a chronic and progressive fibrotic interstitial lung disease characterized by an irreversible decline in lung function and worsening dyspnea. The underlying pathological mechanisms involve resistance to fibroblast apoptosis, epithelial abnormalities, mitochondrial dysfunction, and other interconnected disorders. This disease predominantly affects middle-aged and older individuals, with a higher prevalence observed in men compared to women, and it is associated with a poorer prognosis. The average survival time for patients ranges from 3 to 5 years, with a five-year survival rate between 20 and 40%. Currently, no clinical cure exists for IPF. Treatment primarily involves the use of antifibrotic agents such as pirfenidone and nintedanib to alleviate symptoms and slow disease progression, alongside a focus on palliative care in the advanced stages [1–5]. The incidence and prevalence of IPF have increased in recent years. Based on the most recent meta-analysis available at the time of our literature search, which synthesized epidemiological data through 2023, the projected global incidence of IPF for 2025 was approximately 5.8 per 100,000 individuals, with a prevalence rate of about 17.7 per 100,000 individuals. These figures represent modelled projections derived from pooled historical data rather than direct observations for the year 2025. Notably, the incidence and prevalence rates in North United States are significantly higher than those in Europe and Asia [5]. Although IPF is classified as a rare disease, its progressive nature and high mortality rate impose a substantial burden on patients, their families, and the healthcare system.

Patients with IPF confront not only the risk of irreversible lung function decline and reduced survival but also a significant deterioration in their health-related quality of life (HRQOL) [6]. Consequently, while efforts are made to delay disease progression, a critical clinical objective is to effectively assess and enhance the quality of life for IPF patients. HRQOL, a patient-reported outcome (PRO) [7], provides a comprehensive reflection of disease symptoms, functional status, and psychosocial adaptability. Utilizing patient-reported outcome tools enables the accurate representation of symptoms and quality of life for IPF patients, allowing healthcare providers to assess conditions more holistically, identify potential issues, and develop more personalized treatment plans. Various generic and disease‑specific patient‑reported outcome measures have been developed [7]. These instruments differ substantially in their target populations, content coverage, and measurement properties. For example, some tools focus primarily on physical symptoms and functional limitations, while others incorporate psychosocial and environmental dimensions. The number of items ranges from 5 to 74, and the domains assessed vary considerably across instruments. However, despite this diversity, the measurement properties of these tools have not been systematically compared using a standardized framework, leaving clinicians and researchers uncertain about which instrument is most appropriate for their specific purposes. This methodological gap constitutes the primary rationale for the present study. However, these tools differ considerably in their development background, target populations, dimensions, and measurement properties, leading to inconsistent estimates of quality-of-life impairment in IPF patients [7, 8]. The COSMIN serves as a guideline established by experts in psychology to evaluate clinical measurement tools. Its objective is to identify high-quality assessment instruments for clinicians and researchers, ensuring both scientific rigor and practical applicability [9]. Applying the COSMIN framework to select the most appropriate IPF‑specific quality of life tool is crucial for accurate assessment, personalized interventions, and improved prediction of longitudinal disease trajectories. This systematic review aims to assess existing patient-reported outcome tools designed to measure quality of life in individuals with IPF, adhering to COSMIN guidelines to evaluate their measurement properties. The goal is to provide evidence-based recommendations for the selection of high-quality IPF-specific quality-of-life instruments suitable for future clinical research and practice, based on a comprehensive evaluation of their methodological quality and measurement properties according to COSMIN criteria.

## Methods

### Study design

This systematic review was carried out according to COSMIN (Consensus-based Standards for the selection of health Measurement Instruments) methodology [9]. COSMIN was chosen because it provides a comprehensive framework for evaluating the methodological quality of PROM development and validation studies, rating the quality of each measurement property, and grading the evidence to formulate recommendations for use. In contrast, databases such as PROQOLID (Patient-Reported Outcome and Quality of Life Instruments Database) serve primarily as search sources to identify existing PROMs and validation studies, much like bibliographic databases, and are not designed as methodological assessment tools. Therefore, COSMIN was used as the evaluation framework; PROQOLID, if employed, would only serve as a supplementary search source. These two resources are complementary rather than mutually exclusive in PROM systematic reviews.Although PROQOLID is a valuable resource that provides curated summaries of existing PROMs and is sometimes used as a supplementary source in PROM systematic reviews, we did not employ it as a primary search source in this review. This decision was based on the following considerations: (1) PROQOLID primarily catalogs instrument descriptions and summary information rather than the full-text validation studies required for COSMIN-based methodological quality assessment; (2) the validation studies indexed in PROQOLID are typically retrievable through the bibliographic databases already searched (PubMed, Embase, Web of Science, CINAHL, and Chinese databases); and (3) our search strategy, which incorporated the Terwee methodological filter, was designed to comprehensively capture both instrument development and validation studies. We acknowledge that PROQOLID can serve as a complementary resource, and its omission is a potential limitation discussed in the Limitations section.The manuscript has been written using the PRISMA 2020 reporting guideline and checklist [10] and prospectively registered in PROSPERO (CRD420261286175). The final report will adhere to the PRISMA-COSMIN reporting standards. Any necessary amendments to the protocol will be documented in PROSPERO and reported in the final study report.

### COSMIN systematic review process

**Fig. 1:**
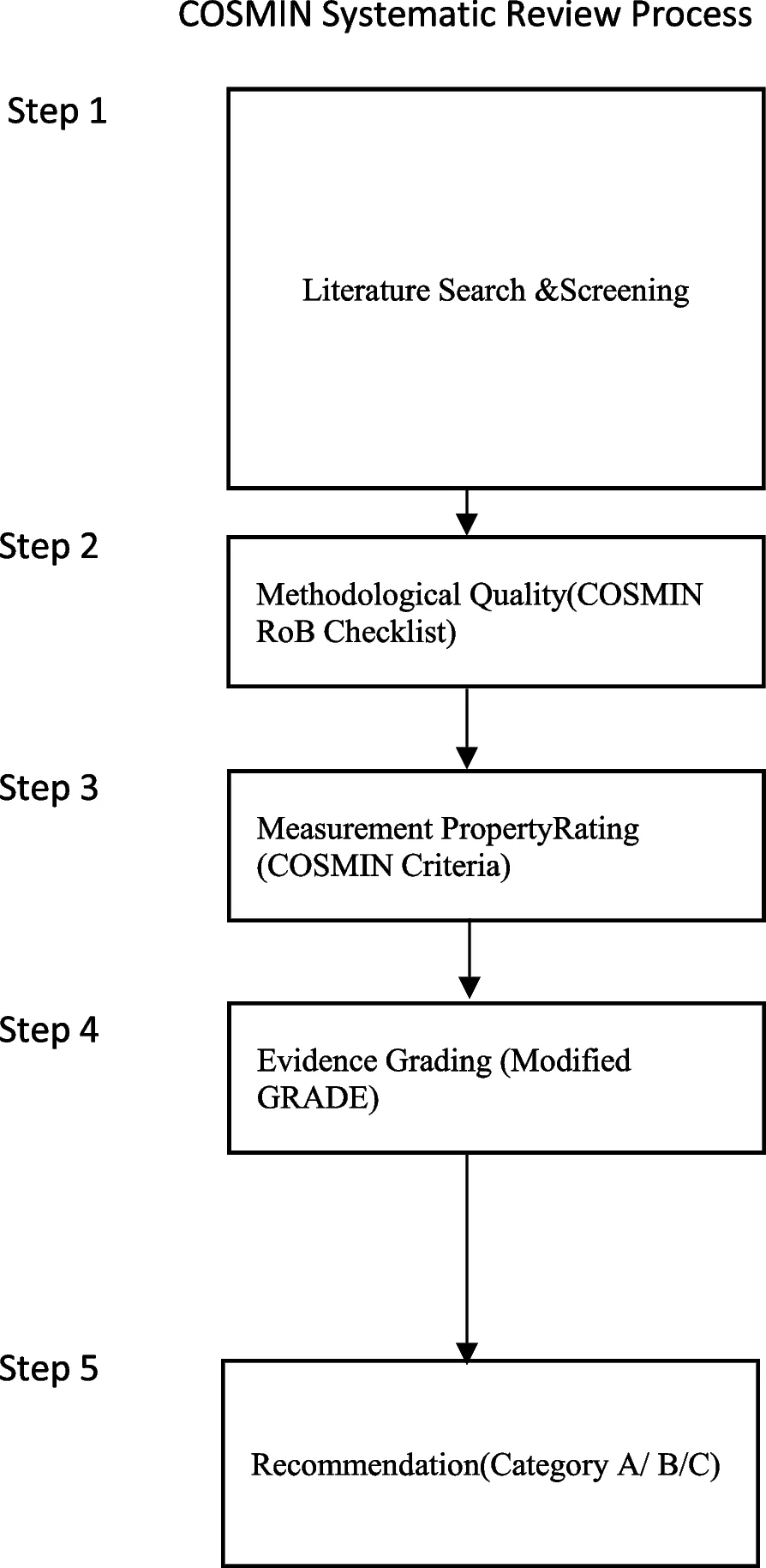
COSMIN systematic review process

The overall evaluation process followed five sequential steps, as illustrated in Fig. 1: (1) literature search and screening; (2) assessment of methodological quality using the COSMIN Risk of Bias Checklist; (3) rating of measurement properties against the COSMIN criteria; (4) grading of the quality of evidence using the modified GRADE approach; and (5) formulation of recommendations.

### Eligibility criteria

Inclusion criteria: ① The study population must consist of patients diagnosed with idiopathic pulmonary fibrosis; ② Participants must be aged 18 years or older; ③ The study must evaluate the measurement properties of an IPF-specific quality-of-life assessment tool; ④ The study must be an original research article (e.g., cross-sectional, cohort, or validation study) evaluating the measurement properties of an IPF-specific quality-of-life instrument.⑤ The study must assess at least one measurement property. Both instrument development studies and validation studies were considered for inclusion. Exclusion criteria: ① The assessment tool is utilized solely as a measure of outcome indicators in surveys or randomized controlled trials; ② The literature constitutes secondary research (systematic review, review); ③ Duplicate publications or overlapping datasets.

### Search strategy (Step 1)

A comprehensive computer search was conducted in PubMed, Embase, Web of Science, CINAHL, CNKI, Wanfang, VIP, and the Chinese Biomedical Literature Database from January 1, 2010 to December 28, 2025. The search combined subject headings and free-text keywords for the target condition (e.g., "Idiopathic Pulmonary Fibrosis," "Usual Interstitial Pneumonia") and measurement instruments (e.g., "quality of life," "questionnaire," "scale"), incorporating the methodological search filter developed by Terwee et al. [11].The search strategy was structured around four key elements: (1) the target population (patients with IPF); (2) the construct of interest (quality of life, health-related quality of life); (3) the types of instruments (questionnaires, scales, patient-reported outcome measures); and (4) the measurement properties of interest (reliability, validity, responsiveness, internal consistency, and other psychometric properties as defined by COSMIN). The search incorporated the methodological filter for measurement properties developed by Terwee et al. [11]. We did not repeat the search at the end of the review, as the search was conducted through December 28, 2025, and the study selection was completed prior to manuscript submission. A representative search strategy for PubMed is provided below (the complete strategies for all databases are available in Appendix 1):

("Idiopathic Pulmonary Fibrosis"[MeSH] OR "Idiopathic Pulmonary Fibrosis"[tiab] OR "Pulmonary Fibrosis, Idiopathic"[tiab] OR "Cryptogenic Fibrosing Alveolitis"[tiab] OR "Usual Interstitial Pneumonia"[tiab] OR "IPF"[tiab]) AND ("Quality of Life"[MeSH] OR "quality of life"[tiab] OR "QOL"[tiab] OR "Health-Related Quality of Life"[tiab] OR "HRQOL"[tiab]) AND ("questionnaire"[tiab] OR "scale"[tiab] OR "instrument"[tiab] OR "tool"[tiab] OR "reliability"[tiab] OR "validity"[tiab] OR "responsiveness"[tiab] OR "psychometric properties"[tiab] OR "measurement error"[tiab] OR "internal consistency"[tiab] OR "factor analysis"[tiab] OR "cross-cultural validity"[tiab] OR "minimal important difference"[tiab]).

The search was limited to publications from January 1, 2010 to December 28, 2025, and restricted to Chinese or English language.The search was limited to publications from January 1, 2010 to December 28, 2025, and restricted to Chinese or English language. In addition to database searching, we manually screened the reference lists of all included full-text articles and relevant review articles to identify any additional eligible studies. Grey literature was not systematically searched.

### Selection of studies

In accordance with the established inclusion and exclusion criteria and search strategy, two researchers (EZ and SM) independently screened the literature and extracted relevant information. In cases of disagreement, a third researcher (JG) was consulted to facilitate discussion and resolution. Duplicate entries were eliminated using NoteExpress 4.2 software. The screening process involved an initial review of titles and abstracts, followed by a comprehensive examination of the full texts, with documents assessed based on the inclusion and exclusion criteria.

### Data extraction

The extracted literature information encompassed the following: author, publication year, scale name, number of scale items and dimensions, scoring method, sample size, study population, and any additional instrument details relevant to the COSMIN assessment.

### Data extraction and methodological quality assessment

Four researchers (EZ, SM, SW, and YD) independently assessed all included tools according to COSMIN guidelines [9]. Prior to the consensus discussion, the inter-rater reliability across the four reviewers, assessed by Fleiss ‘ kappa, was 0.88 (95% CI: 0.82–0.94), indicating almost perfect agreement. Disagreements were resolved through discussion; if consensus could not be reached, the corresponding author (JG) made the final decision. After the discussion sessions, the final agreement reached 95%. The evaluation covered three components: methodological quality, measurement property quality, and evidence grading. We applied two COSMIN tools: ① COSMIN Risk of Bias Checklist [12]: which evaluates the methodological quality of studies on each measurement property (including PROM development). Each item is scored as "very good" (V), "adequate" (A), "doubtful" (D), or "inadequate" (I). Following the COSMIN "worst score counts" principle, the overall rating for each domain is determined by the lowest score assigned to any item within that domain (i.e., if one item in a domain is rated "inadequate, " the entire domain receives an "inadequate" rating, regardless of other item scores). ② COSMIN quality criteria [9]: which assess the results of each measurement property as "sufficient" (+), "insufficient" (−), or "indeterminate" (?).Both tools address the same set of measurement properties: content validity, structural validity, internal consistency, cross‑cultural validity/measurement invariance, reliability, measurement error, criterion validity, hypothesis testing for construct validity, and responsiveness. The Risk of Bias checklist additionally covers PROM development.

### Grading of quality of evidence

The quality of evidence was graded using the modified GRADE approach as recommended by the COSMIN guideline [9]. According to this approach, the evidence from each included study is initially assumed to be of high quality and is then downgraded based on four criteria: risk of bias, imprecision, inconsistency, and indirectness. This process resulted in four quality grades: "high," "moderate," "low," and "very low." The modified GRADE approach applied in this study follows the COSMIN v2.0 protocol [9]; for additional methodological illustration, we also referred to the Chinese-language adaptation described by Chen et al. [13]. Initially, each study included in the analysis was classified as “high quality” before being downgraded based on four criteria: risk of bias, imprecision, inconsistency, and indirectness. This process resulted in four quality grades: “high”, “moderate”, “low”, and “very low.” The research team then discussed and decided the final recommendation category.The recommendation categories are defined as follows: Category A indicates that content validity and internal consistency are both deemed “sufficient”; Category B encompasses studies that do not fit into Categories A or C; and Category C signifies that high-quality evidence demonstrates that the measurement properties are “inadequate” [13].According to the COSMIN framework [9, 13], the three recommendation categories are defined in detail as follows:Category A (recommended for use): The PROM has sufficient evidence for content validity (any level of evidence) and sufficient evidence for internal consistency (at least low-quality evidence). PROMs in this category are considered to have credible results and can be recommended for use in clinical practice and research.Category B (promising but needs further validation): The PROM does not meet the criteria for Category A and does not have high-quality evidence showing insufficient measurement properties. These PROMs have potential for application but require additional validation studies to confirm their measurement properties.Category C (not recommended): High-quality evidence demonstrates that at least one measurement property of the PROM is insufficient. These PROMs are not recommended for use until further evidence supports their validity [13].

## Results

**Fig. 2:**
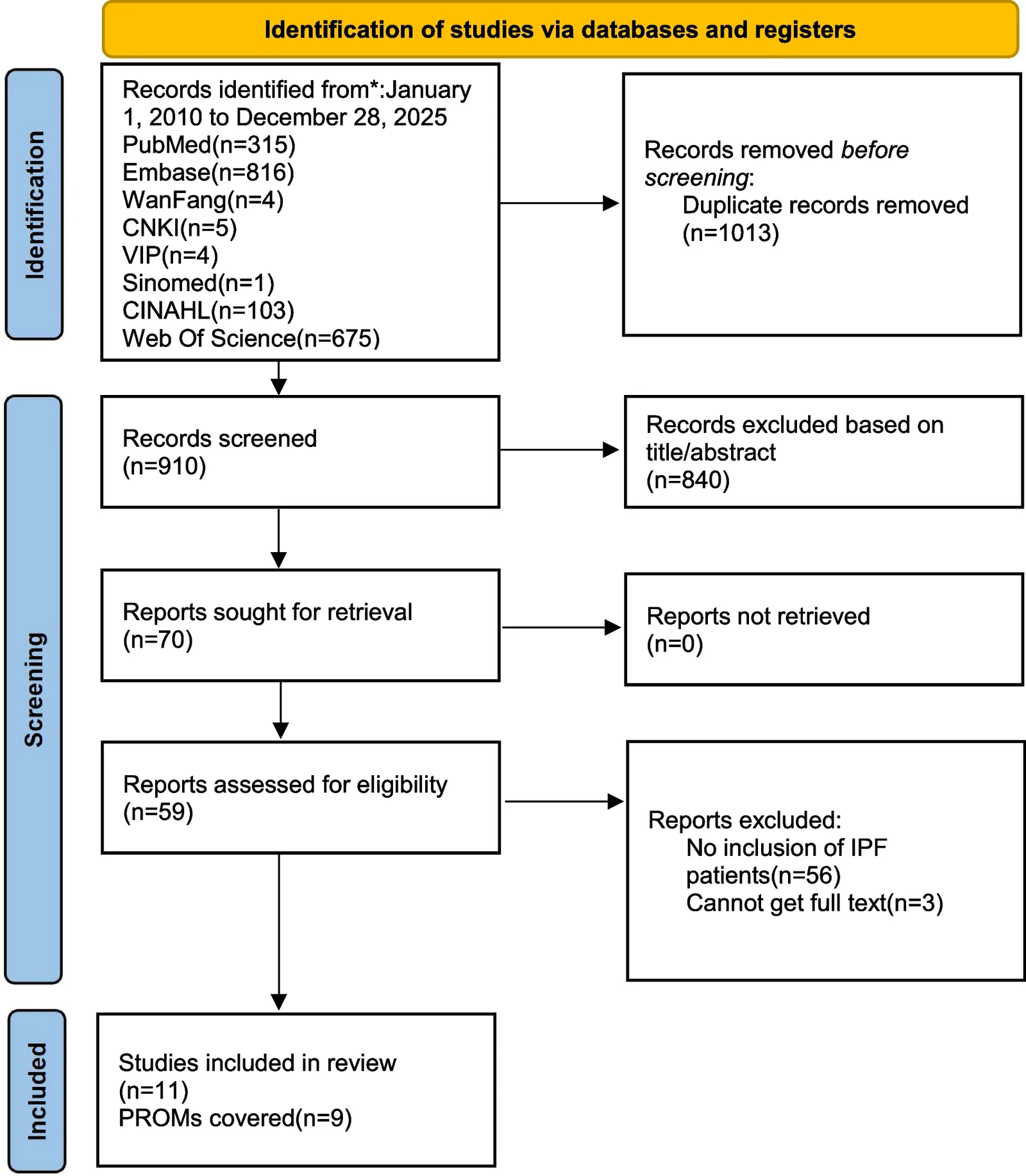
PRISMA 2020 flow diagram

A preliminary search identified 1923 records (6 in Chinese and 1917 in English). After removing duplicates, 910 records remained. Of these, 840 were excluded based on titles and abstracts, leaving 70 full texts for assessment.Ultimately, 11 documents [14–24] met the inclusion criteria.The reasons for exclusion at the title/abstract screening stage included: (1) studies not focused on IPF-specific quality-of-life instruments; (2) studies using instruments only as outcome measures in clinical trials without psychometric evaluation; (3) duplicate publications; and (4) review articles, conference abstracts, or non-original research. For the 11 full-text articles that could not be retrieved, we attempted to contact the corresponding authors via email; however, we did not receive responses, and these articles were therefore excluded. The screening process is shown in Fig. 2.

### Basic characteristics of included studies

The 11 included studies included 9 tools, including the A Tool to Assess Quality of Life in IPF (ATAQ-IPF), the Chinese version of ATAQ-IPF (cATAQ-IPF), Idiopathic Pulmonary Fibrosis Traditional Chinese Medicine Health-Related Quality of Life (IPF-TCM-HRQOL32), the Living with Idiopathic Pulmonary Fibrosis Questionnaire (L-IPF), Quality of life questionnaire for fibrosing lung diseases (QPF), R-Scale-PF for Pulmonary Fibrosis (R-Scale-PF), an IPF-specific version of the St. George's Respiratory Questionnaire (SGRQ-IPF), Derived version of the St. George's Respiratory Questionnaire for Idiopathic Pulmonary Fibrosis (SGRQ-Ider), Patient-Reported Outcome Scale for Idiopathic Pulmonary Fibrosis (IPF-PRO). The general information table of included literature is shown in Table 1.

**Table 1:** Characteristics of included literature

Author	Year	Country	PROMS	sample size	items/domains	Target population
Swigris et al. [16]	2010	United States	ATAQ-IPF	95	74/13	patients with IPF
Zang et al. [24]	2016	China	IPF-TCM-HRQOL32	50	32/4	patients with IPF
Ruili PAN [23]	2014	China	cATAQ-IPF	92	74/13	patients with ILD and patients with IPF
Pan et al. [17]	2019	China	cATAQ-IPF	92	74/13	patients with ILD and patients with IPF
Swigris et al. [14]	2020	United States	L-IPF	125	35/5	patients with IPF
Kirsten et al. [19]	2021	Germany	QPF	200	23/5	patients with IPF and patients with NIP
Scallan et al. [15]	2021	United States	R-Scale-PF	100	5/5	patients with IPF
Yorke et al. [22]	2010	Britain	SGRQ-I	158	34/3	patients with IPF
Prior et al. [20]	2019	Denmark	SGRQ-I	150	34/3	patients with IPF
Prior et al. [21]	2021	Denmark	SGRQ-I_der_	150	34/3	patients with IPF
Yang Xie et al. [18]	2024	China	IPF-PRO	180	18/4	patients with IPF

Measurement properties (Steps 2 & 3)

None of the 11 included studies explicitly reported measurement error as a distinct measurement property. However, it should be noted that five studies [14, 17, 20, 21, 23] reported test–retest reliability with ICC values and could have potentially calculated SEM or MDC from their data; these calculations were not performed or reported in the original publications. The remaining six studies [15, 16, 18, 19, 22, 24] did not include test–retest designs and therefore lacked the primary data required for SEM or MDC calculation. Thus, the absence of measurement error reporting reflects a genuine absence of calculation in the primary studies, rather than a failure to report existing calculations in COSMIN-compatible categories. The methodological quality and measurement properties for all other attributes are summarized in Table 2.

**Table 2:** Methodological quality and measurement properties of assessment tools

PROM	PROM development	Content validity			Structural validity		Internal consistency		Cross-cultural validity\measurement invariance	
		Relevance	Comprehensiveness	Comprehensibility	index	Results	Cronbach’s α	results	index	results
ATAQ-IPF [16]	D	D/?	D/?	D/?	Rasch analysis	A/?	0.51 ~ 0.83	V/-	NR	NR
cATAQ-IPF [17, 23]	NA	D/?	D/?	D/?	NR	NR	0.60 ~ 0.95	V/-	Not reported in detail	D/?
IPF-TCM-HRQOL32 [24]	D	D/?	D/?	D/?	factor analysis	D/?	0.88 ~ 0.97	V/+	NR	NR
L-IPF [14]	D	D/?	D/?	D/?	factor analysis	I/+	0.93 ~ 0.98	V/+	NR	NR
QPF [19]	D	NR	NR	NR	NR	NR	0.30 ~ 0.88	V/-	NR	NR
R-Scale-PF [15]	D	D/?	D/?	D/?	NR	NR	0.825	V/+	NR	NR
SGRQ-I [22]	I	I/-	I/-	I/-	Rasch analysis	A/?	0.62 ~ 0.86	V/-	NR	NR
SGRQ-I [20]	NA	NR	NR	NR	NR	NR	0.92	V/+	NR	NR
SGRQ-I_der_ [21]	NA	NR	NR	NR	NR	NR	0.74 ~ 0.86	V/+	Not reported in detail	D/?
IPF-PRO [18]	D	D/?	D/?	D/?	factor analysis	V/?	0.75 ~ 0.88	V/+	NR	NR

PROM	Reliability		Measurement error		Criterion validity		Hypothesis testing for construct validity		Responsiveness	
	ICC	results	assess index	results	index	results	assess method	results	assess method	results
ATAQ-IPF [16]	NR	NR	NR	NR	not compared with gold standard	I/-	Perform subgroup analysis	V/+	NR	NR
cATAQ-IPF [17, 23]	0.89 ~ 0.98	A/+	NR	NR	not compared with gold standard	I/-	Perform subgroup analysis	V/?	NR	NR
IPF-TCM-HRQOL32 [24]	NR	NR	NR	NR	NR	NR	NR	NR	NR	NR
L-IPF [14]	0.79 ~ 0.91	V/+	NR	NR	not compared with gold standard	I/-	1 comparison scale	V/+	NR	NR
QPF [19]	NR	NR	NR	NR	NR	NR	1 comparison scale	V/-	Perform subgroup analysis	A/+
R-Scale-PF [15]	NR	NR	NR	NR	not compared with gold standard	I/-	2 comparison scale	V/+	NR	NR
SGRQ-I [22]	NR	A/+	NR	NR	not compared with gold standard	I/-	Perform subgroup analysis and compared with 5 scales	A/+	NR	NR
SGRQ-I [20]	0.92	A/+	NR	NR	NR	NR	Perform subgroup analysis and compared with other instruments	A/+	NR	NR
SGRQ-I_der_ [21]	0.99	A/+	NR	NR	NR	NR	Perform subgroup analysis	V/+	Compare with other scales	V/+
IPF-PRO [18]	NR	NR	NR	NR	not compared with gold standard	I/-	Perform subgroup analysis	V/?	NR	NR

*ATAQ-IPF* A Tool to Assess Quality of Life in IPF, *cATAQ-IPF* the Chinese version of ATAQ-IPF, *IPF-TCM-HRQOL32* Idiopathic Pulmonary Fibrosis Traditional Chinese Medicine Health-Related Quality of Life, *L-IPF* the Living with Idiopathic Pulmonary Fibrosis Questionnaire, *QPF* Quality of life

questionnaire for fibrosing lung diseases, *R-Scale-PF* R-Scale-PF for Pulmonary Fibrosis, *SGRQ-IPF* an IPF-specific version of the St. George's Respiratory Questionnaire, *SGRQ-Ider* Derived version of the St. George's Respiratory Questionnaire for Idiopathic Pulmonary Fibrosis, *IPF-PRO* Patient-Reported Outcome Scale for Idiopathic Pulmonary Fibrosis

Overall rating: + (sufficient);—(insufficient); ± (inconsistent);? (indeterminate)

Methodological quality: V (very good); A (adequate); D (doubtful); I (inadequate); NA (not applicable); NR (not reported)

### Development of PROMs

The two studies [17, 23] involved cross‑cultural translation of existing scales and were therefore not applicable for PROM development evaluation. The remaining studies all had notable limitations in this domain, receiving ratings of "doubtful" or "inadequate." Common issues included: lack of pre‑interviews or pilot testing; reliance on experts and literature rather than patient input for item generation; unclear reporting on whether interviews were recorded and transcribed verbatim; lack of independent data analysis by two or more researchers; and absence of cognitive interviews to assess patient comprehensibility.

### Content validity

Eight studies [14–18, 22–24] assessed content validity. Among these, three studies [14, 16, 18] employed structured or semi-structured interviews with patients and engaged focus groups to evaluate the content validity of the scale. One study [24] utilized expert consultation to assess content validity, while two studies [17, 23] relied on expert meetings for the same purpose. Additionally, one study [15] conducted semi-structured interviews to gather patient perspectives on content validity. None of the aforementioned seven studies specified whether data analysis was performed by two or more researchers, whether the interviews were transcribed verbatim, or whether experienced interviewers or moderators were involved. Due to the doubtful description of the research processes, the methodological quality was deemed “doubtful,” and the measurement attributes were rated as “?”. In contrast, one study [22] exclusively employed Rasch analysis to screen items without consulting patients or experts, resulting in a methodological quality rating of “inadequate” and measurement attributes of “-”.

### Structural validity

Five studies reported on Structural validity [14, 16, 18, 22, 24], of which two studies [16, 22] employed item response theory (IRT). Due to a sample size of fewer than 200 participants, the methodological quality was deemed “adequate,” and the lack of reported local independence resulted in the measurement properties being rated as “?”. One study [24] utilized classical test theory (CTT) but conducted only exploratory factor analysis with an inadequate sample size, leading to a methodological quality rating of “doubtful” and measurement properties rated as “?”. Another study [18] applied classical test theory and performed both exploratory and confirmatory factor analyses; however, the relevant indicators did not satisfy the “ + ” requirement, resulting in a methodological quality rating of “very good” and measurement attributes rated as “?”. Finally, one study [14] also used classical test theory with exploratory and confirmatory factor analyses, but the sample size fell short of the qualification standard, despite the relevant indicators meeting the “ + ” requirements, leading to a methodological quality rating of “inadequate” and measurement attributes rated as “ + ”.

### Internal consistency

All 11 studies [14–24] reported internal consistency and calculated Cronbach’s α coefficient, indicating a “very good” methodological quality. Among these, six studies [16, 17, 19, 20, 22, 23] presented scales with a Cronbach’s α coefficient of less than 0.7 in one or more dimensions. Consequently, the measurement properties of these six studies are rated as “-”, while the remaining studies are all rated as “ + ”.

Cross-cultural validity/Measurement invariance.

Two studies [17, 20] demonstrated cross-cultural validity; however, the data analysis methods employed were unclear, and neither study performed multi-group factor analysis. Consequently, the methodological quality was deemed “doubtful, “ and the measurement properties remained unspecified.

### Test − retest reliability

Five studies [14, 17, 20, 21, 23] reported Test − retest reliability. Among these, four studies [14, 17, 20, 21] adhered to the two-week retest interval recommended by the COSMIN guideline. It can be inferred that the measurement conditions for patients were consistent, the patients' conditions remained stable, and the methodological quality evaluations were classified as “very good” and “adequate.” In contrast, one study [23] established a test–retest interval exceeding two weeks without providing adequate justification, resulting in a methodological quality rating of “ inadequate.” Notably, none of the five studies reporting test − retest reliability exhibited an intraclass correlation coefficient (ICC) lower than 0.7, indicating that all measurement properties were rated as “ +.”

### Criterion validity

Criterion validity was assessed in five studies [14, 15, 18, 22, 23]. According to the COSMIN framework, criterion validity for PROMs is applicable only when a "gold standard" exists for the construct being measured; the one exception is when a shortened version of a PROM is compared against its original long version, in which case the original version can serve as the gold standard. In all five studies, no such gold standard was available, and the authors instead compared the IPF-specific tools with other commonly used scales (e.g., SGRQ, EQ-5D, or K-BILD) that were not established as gold standards for IPF-specific quality of life. Consequently, all five studies were rated as "inadequate" in methodological quality for criterion validity. None of the studies assessed predictive criterion validity; all comparisons were concurrent.

### Hypothesis testing for construct validity

Ten studies [14–23] investigated the hypothesis testing for construct validity. Five studies [16–18, 21, 23] assessed structural validity through subgroup comparisons. Each of these studies detailed the significant characteristics of the subgroups, resulting in a methodological quality rating of “very good” or “adequate.” In three studies [17, 18, 23], the authors did not define hypothesis, leading to an indeterminate measurement property rating of “?,” while the remaining two studies [16, 21] received a rating of “ +.” Three studies [14, 15, 19] conducted hypothesis testing for construct validity by comparing with other measurement tools, achieving a methodological quality rating of “very good” or “adequate.” Notably, the results of one study [19] were inconsistent with the hypothesis, resulting in a measurement attribute rating of “-”, whereas the other two studies [14, 15] received a rating of “ +.” Additionally, two studies [20, 22] simultaneously compared other measurement tools and conducted subgroup comparisons, resulting in a methodological quality rating of “adequate” and a measurement property rating of “ +.”

### Responsiveness

Two studies [19, 21] evaluated responsiveness. One study [21] assessed responsiveness by comparing it with other measurement tools. Although the tools employed demonstrated adequate measurement properties, some were not specifically designed for patients with idiopathic pulmonary fibrosis. Consequently, the methodological quality was rated as “very good” and “adequate.” Another study [19] measured response through subgroup comparisons, reporting the most significant characteristics of these subgroups; thus, its methodological quality was rated as “adequate.” The findings from both studies align with the hypothesis, indicating that the measurement properties are rated as “ +.”

### Summary of findings (Steps 4 & 5)

The evaluation of the evidence level for the assessment tool, based on the modified version of COSMIN GRADE, was conducted across four dimensions: risk of bias, imprecision, inconsistency, and indirectness. Detailed information is provided in Table 3.

**Table 3:** Evidence levels and recommendations for inclusion in assessment tools

PROM	Quality of evidence									Recommendation
	Content validity	Structural validity	Internal consistency	Cross-cultural validity\measurement invariance	Reliability	Measurement error	Criterion validity	Hypothesis testing for construct validity	Responsiveness	
ATAQ-IPF [16]	Moderate	Moderate	Moderate	NR	NR	NR	Low	Moderate	NR	B
cATAQ-IPF [17, 23]	Moderate	NR	Moderate	Moderate	Moderate	NR	Very Low	Moderate	NR	B
IPF-TCM-HRQOL32 [24]	Moderate	Moderate	Moderate	NR	NR	NR	NR	NR	NR	B
L-IPF [14]	Moderate	Moderate	Moderate	NR	NR	NR	Low	Moderate	NR	B
QPF [19]	NR	NR	High	NR	NR	NR	NR	High	High	B
R-Scale-PF [15]	Low	NR	High	NR	NR	NR	Low	High	NR	B
SGRQ-I [22]	Moderate	Moderate	Moderate	NR	High	NR	Low	High	NR	B
SGRQ-I [20]	NR	NR	High	NR	NR	NR	NR	High	NR	B
SGRQ-Ider [21]	NR	NR	High	Moderate	High	NR	NR	High	High	B
IPF-PRO [18]	Moderate	High	High	NR	High	NR	Low	High	NR	B

Grading QOE: High. Moderate; Low; Very low according to GRADE approach modified

Risk of bias:The methodological quality of eight articles [14–18, 22–24] was deemed “doubtful” regarding content validity, resulting in a downgrade of one level. One article [14] exhibited “doubtful” structural validity and was similarly downgraded by one level. The remaining studies were classified as “very good” or “adequate,” and thus were not downgraded. The methodological quality for internal consistency of eleven articles [14–24] was rated as “very good,” and these studies will not be downgraded. Two studies [17, 20] displayed “doubtful” methodological quality concerning cross-cultural validity and will be downgraded by one level. One study [23] will be downgraded by two levels due to “ inadequate” test − retest reliability in methodological quality; however, its follow-up study has corrected the retest interval, preventing an overall downgrade. The remaining four articles [14, 17, 20, 21] are classified as “adequate” and will not be downgraded. The methodological quality of five studies [14, 15, 18, 22, 23] concerning criterion validity is rated as “inadequate” and will be downgraded by two levels. The methodological quality for hypothesis testing of ten studies [14–23] regarding structural validity is classified as “very good” or “adequate,” and therefore will not be downgraded. Additionally, the methodological quality of two studies [14–18, 22–24] is rated as “very good” or “adequate,” so these will also not be downgraded.

#### Imprecision

According to the COSMIN–GRADE criteria, evidence was downgraded for imprecision based on sample size. Four studies [16, 17, 23, 24] included 50–99 participants and were therefore downgraded by one level. The remaining seven studies had sample sizes of 100 or more and were not downgraded for imprecision. No included study had a sample size of fewer than 50 participants; thus, no study was downgraded by two levels for imprecision.”

#### Inconsistency

No unexplained inconsistencies were found in the scales included in this article.

#### Indirectness

The majority of research subjects in this article were patients with idiopathic pulmonary fibrosis. Two studies [17, 23] included non-IPF patients, resulting in a certain degree of indirectness; thus, these studies were downgraded by one level. One study [19] included only a small number of patients with non-specific interstitial pneumonia, and the degree of indirectness was deemed acceptable, so it was not downgraded. Considering these factors, all assessment tools were downgraded and evaluated. There was insufficient evidence to establish the content validity an internal consistency of all tools as “adequate,” leading to their classification as Category B recommendations.

## Discussion

The methodological quality and measurement properties of patient-specific quality of life scales for idiopathic pulmonary fibrosis require enhancement. Only a limited number of scales are utilized internationally to assess the quality of life specific to patients with IPF, and the overall methodological quality and measurement properties of the nine included tools remain inadequate. The key finding of this review is that although nine IPF-specific quality-of-life instruments are available, none met the COSMIN criteria for Category A recommendation, primarily due to insufficient evidence for content validity and internal consistency. The IPF-PRO scale showed comparatively stronger evidence for several measurement properties but remains provisionally recommended pending further validation.

First, regarding the development of patient-reported outcome measures (PROMs) and content validity, most studies do not adequately describe the specific qualitative research methods employed or the content validity verification processes. For instance, it is often unclear whether cognitive interviews or pre-experiments were conducted, whether the interview outline was appropriate, whether the content was transcribed verbatim, whether the interviews were facilitated by experienced moderators, and whether at least two researchers independently analyzed the data. These methodological shortcomings directly impact the quality of the scale's content validity. Consequently, future scale preparation processes should include a detailed description of the specific research steps to enhance transparency. Furthermore, while content validity is the most critical measurement attribute [25] of a measurement tool, the studies included in this article fail to report content validity comprehensively. In terms of item screening and other considerations, most studies prioritize expert opinions while neglecting the perspectives of research subjects, leading to incomplete reporting of content validity results. The end users of the assessment tools are the research subjects. Future research should employ systematic qualitative methods, such as cognitive interviews, to gather feedback and insights from these subjects, thereby enhancing the methodological quality of the content validity of the assessment tools and the comprehensiveness of the reports.In particular, the IPF‑TCM‑HRQOL32 scale lacked data on test–retest reliability, criterion validity, hypothesis testing, and responsiveness. Among the nine tools reviewed, it had the most limited measurement evidence. This substantial gap limits confidence in its applicability and underscores the urgent need for comprehensive validation before any clinical use can be considered.

It is advisable that, when developing and compiling new scales, exploratory factor analysis be utilized to delineate the factor structure, followed by confirmatory factor analysis to assess the fit indices, with relevant metrics reported in detail. Adequate verification groups should be included to increase the sample size and improve the measurement properties of structural validity.

The COSMIN Guidelines recommend that the cross-cultural validity [26] of a measurement tool can be effectively evaluated by analyzing its measurement invariance or item functional differences. When conducting future cross-cultural adaptations, researchers should analyze differences in item functions or evaluate multiple groups of factors across similar groups in two distinct samples. They must report the measurement and evaluation processes in detail and ensure an adequate sample size to enhance the quality of cross-cultural validity reporting.

Regarding test − retest reliability, the COSMIN guidelines recommend a test–retest reliability interval of approximately 2 weeks. Any deviation from this interval should be accompanied by a suitable explanation [27]. Unless there are compelling reasons for deviations in future test − retest reliability testing, a 2-week retest interval should be maintained to safeguard methodological quality and measurement properties.

Measurement error contributes to variation among subjects beyond true variation [27]. Future studies focused on developing or validating IPF-specific quality of life assessment scales should enhance measurement error reporting to clarify the clinical applicability of these tools.

Regarding criterion validity, the COSMIN guidelines suggest that, in the absence of a definitive "gold standard" for assessing quality of life in patients with IPF, the original version of the tool should be regarded as the "gold standard" when comparing the shortened version with the original tool [10]. Consequently, it is advisable for future research to adhere to the COSMIN guidelines and utilize the original scale as the preferred gold standard. In the absence of a gold standard, a scale with comparable measurement content and superior methodological quality may serve as an alternative, complemented by multidimensional verification through structural validity evidence to enhance the reliability of the assessment results [9].

Selection and recommendation of specific quality of life assessment scales for patients with idiopathic pulmonary fibrosis.

The nine measurement tools included in this study are all classified as Category B recommendations and possess certain potential; however, further research is necessary to assess their quality. Among these tools, the IPF‑PRO scale demonstrated several advantages that support its provisional recommendation. First, it was developed specifically for Chinese IPF patients, ensuring linguistic and cultural appropriateness, whereas most other tools were developed in Western populations and would require cross‑cultural adaptation for use in China. Second, with only 18 items organized into four clinically meaningful dimensions (physiology, psychology, environment, and satisfaction), the scale is concise and feasible for routine clinical use, reducing patient burden. Third, it employs a 5‑point Likert scale, a familiar format that facilitates patient comprehension and reliable self‑reporting. Fourth, it showed high‑quality evidence for internal consistency, structural validity, and hypothesis testing—three properties essential for a reliable and valid measure. However, measurement error and responsiveness have not yet been tested for the IPF‑PRO scale. These gaps should be addressed in future longitudinal studies to further substantiate its clinical utility.When the same tool was evaluated in multiple studies, occasional inconsistencies were observed. For example, two studies of the SGRQ‑I scale [20, 22] both used Rasch analysis and reported adequate methodology, but structural validity remained indeterminate due to failure to report local independence, and internal consistency varied across dimensions. In such cases, greater weight was given to larger samples and more rigorous methodology. Nevertheless, inconsistency across studies reduced the certainty of evidence and contributed to the tool's final classification.It should also be noted that several tools achieved "High" evidence grades for hypothesis testing yet remained classified as Category B. According to the COSMIN framework, a Category A recommendation requires sufficient content validity and internal consistency, both supported by at least moderate-quality evidence. None of the nine tools met both criteria concurrently. Even when other properties, such as construct validity or reliability, showed high-quality evidence, the failure to establish content validity with sufficient rigor precluded a Category A classification.This underscores the primacy of content validity in the COSMIN framework and highlights a critical area for improvement in future IPF-specific PROM development.

Strengths and limitations of individual tools.

Several tool‑specific strengths and limitations merit further discussion. ATAQ‑IPF and its Chinese version (cATAQ‑IPF) are among the earliest IPF‑specific tools and cover a broad range of domains with cross‑cultural adaptation, yet their internal consistency fell below acceptable thresholds in several dimensions and content validity evidence was limited. L‑IPF was developed with relatively rigorous patient input and demonstrated strong internal consistency and test–retest reliability, but its structural validity was assessed with an insufficient sample and criterion validity was lacking. Both versions of SGRQ‑I showed high test–retest reliability and good hypothesis testing performance, with the derived version also demonstrating acceptable responsiveness; however, content validity reporting was inadequate and cross‑cultural evidence remains incomplete. IPF‑PRO, as discussed, has notable strengths in cultural specificity for Chinese patients, brevity, and robust evidence for several key psychometric properties, although measurement error and responsiveness have not yet been evaluated. Finally, IPF‑TCM‑HRQOL32 is the only tool that integrates TCM concepts into quality‑of‑life measurement for IPF patients, but its validation evidence is the most limited, with most measurement properties unreported and comprehensive COSMIN‑based validation still required. Collectively, these observations highlight the need for more rigorous and complete psychometric evaluation across all existing IPF‑specific tools.It should be emphasized that, according to the COSMIN framework, a Category A recommendation requires sufficient evidence for content validity (any level) and at least low-quality evidence for sufficient internal consistency. Since none of the nine tools met both criteria simultaneously, a formal Category A recommendation cannot be made. The provisional recommendation of the IPF-PRO scale is therefore offered as a supplementary clinical guidance based on: (1) its comparatively stronger evidence for structural validity, internal consistency, and hypothesis testing; and (2) practical considerations including cultural specificity for Chinese patients, brevity (18 items), and feasibility for routine use. This provisional recommendation should not be equated with a COSMIN Category A endorsement, and its use should be accompanied by awareness of its unvalidated measurement properties.

### Limitations of this study

First, this review exclusively examined Chinese and English literature from six databases, potentially resulting in the omission of certain tools. To mitigate this potential limitation, we manually screened the reference lists of all included studies and relevant reviews to identify additional eligible studies; however, relevant instruments published exclusively in grey literature or in languages other than Chinese or English may still have been missed. Second, the COSMIN evaluation process depends on the evaluator, introducing a degree of subjectivity that may lead to bias.Third, although we searched four Chinese-language databases (CNKI, Wanfang, VIP, and CBM), only six of the 1,923 initially identified records were in Chinese. This asymmetry may reflect several factors: (1) the limited number of IPF-specific quality-of-life instruments developed and validated in Chinese populations; (2) potential deficiencies in our Chinese-language search strategies, including the selection and combination of Chinese keywords; or (3) the possibility that relevant Chinese-language validation studies were published in Chinese journals not indexed in the databases we searched. While the small number of Chinese records may also suggest that the IPF-PRO scale [18] and IPF-TCM-HRQOL32 [24] represent the primary instruments developed in the Chinese context, we acknowledge that our Chinese-language search strategy may have been suboptimal and that relevant studies could have been missed.

## Conclusion

Based on the COSMIN-based evaluation of the nine identified IPF-specific quality-of-life instruments, none met the criteria for Category A recommendation; all were classified as Category B. Among these, the IPF-PRO scale is thus recommended with caution, pending further validation of its unassessed properties.. A diverse array of tools is currently employed internationally to evaluate the quality of life in IPF patients; however, most exhibit deficiencies in methodological quality and measurement properties. Notably, there is often inadequate or inconsistent reporting regarding content validity, cross-cultural validity, measurement error, and criterion validity. Among the nine assessment tools reviewed, none achieved the Category A recommendation standard, while all were classified as Category B recommendations. Following a thorough comparison, the IPF-PRO scale is provisionally recommended for use in Chinese clinical settings, with the understanding that its structural validity results were rated as "indeterminate" (despite high methodological quality and a "High" GRADE rating), content validity is supported by moderate-quality evidence, and criterion validity is supported by low-quality evidence. Future research should aim to assess and enhance the unreported measurement properties of existing tools (particularly measurement error and responsiveness for the IPF-PRO), adhere to COSMIN guidelines in the design and development of new scales, and ultimately provide patients with high-quality and accurate assessment instruments.

## Supplementary Information

Supplementary Material 1.

## Data Availability

All data generated or analysed during this study are included in this published article.

## References

[1] Yu QY, Tang XX. Irreversibility of pulmonary fibrosis. Aging Dis. 2022;13:73–86. 10.14336/AD.2021.0730. 35111363 PMC8782547

[2] van Manen MJG, Geelhoed JJM, Tak NC, et al. Optimizing quality of life in patients with idiopathic pulmonary fibrosis. Ther Adv Respir Dis. 2017;11:157–69. 10.1177/1753465816686743. 28134007 PMC5933652

[3] Cruwys S, Hein P, Humphries B, et al. Drug discovery and development in idiopathic pulmonary fibrosis: the changing landscape. Drug Discov Today. 2024;29:104207. 10.1016/j.drudis.2024.104207. 39396672

[4] Martinez FJ, Collard HR, Pardo A, et al. Idiopathic pulmonary fibrosis. Nat Rev Dis Primers. 2017;3:17074. 10.1038/nrdp.2017.74. 29052582

[5] Golchin N, Patel A, Scheuring J, et al. Incidence and prevalence of idiopathic pulmonary fibrosis: a systematic literature review and meta-analysis. BMC Pulm Med. 2025;25:378. 10.1186/s12890-025-03836-1. 40775309 PMC12330001

[6] Nolan CM, Birring SS. Promising developments in IPF patient-reported outcome measures. Eur Respir J. 2022;59:2102312. 10.1183/13993003.02312-2021. 35027375

[7] Zhao RM, Dai HP, Wang C. Patient-reported outcomes in idiopathic pulmonary fibrosis: health-related quality of life scales[in Chinese]. Chin J Pract Intern Med. 2023;43:155–8. 10.19538/j.nk2023020114.

[8] Swigris J, Olson AL, Brown K. Understanding and optimizing health-related quality of life and physical functional capacity in idiopathic pulmonary fibrosis. Patient Relat Outcome Meas. 2016;7:29–35. 10.2147/PROM.S74857. 27274328 PMC4876092

[9] Mokkink LB, Elsman EBM, Terwee CB. COSMIN guideline for systematic reviews of patient-reported outcome measures version 2.0. Qual Life Res. 2024;33:2929–39. 10.1007/s11136-024-03761-6. 39198348 PMC11541334

[10] Page MJ, McKenzie JE, Bossuyt PM, et al. The PRISMA 2020 statement: an updated guideline for reporting systematic reviews. BMJ. 2021;372:n71. 33782057 10.1136/bmj.n71 PMC8005924

[11] Terwee CB, Jansma EP, Riphagen II, et al. Development of a methodological PubMed search filter for finding studies on measurement properties of measurement instruments. Qual Life Res. 2009;18:1115–23. 10.1007/s11136-009-9528-5. 19711195 PMC2744791

[12] Mokkink LB, de Vet HCW, Prinsen CAC, et al. COSMIN risk of bias checklist for systematic reviews of patient-reported outcome measures. Qual Life Res. 2018;27:1171–9. 10.1007/s11136-017-1765-4. 29260445 PMC5891552

[13] Chen YT, Shen LJ, Peng J, et al. Modified GRADE evaluation of COSMIN method for patient reported outcome measure. J Nurs PLA. 2020;37:57–60.

[14] Swigris JJ, Andrae DA, Churney T, et al. Development and initial validation analyses of the living with idiopathic pulmonary fibrosis questionnaire. Am J Respir Crit Care Med. 2020;202:1689–97. 10.1164/rccm.202002-0415OC. 32634038 PMC7737580

[15] Scallan C, Strand L, Hayes J, et al. R-scale for pulmonary fibrosis: a simple, visual tool for the assessment of health-related quality of life. Eur Respir J. 2022;59:2100917. 10.1183/13993003.00917-2021. 34112729

[16] Swigris JJ, Wilson SR, Green KE, et al. Development of the ATAQ-IPF: a tool to assess quality of life in IPF. Health Qual Life Outcomes. 2010;8:77. 10.1186/1477-7525-8-77. 20673370 PMC2920246

[17] Pan R, Swigris JJ, Zhao Y, et al. Reliability and validity of Chinese version of a tool to assess the quality of life in idiopathic pulmonary fibrosis in patients with interstitial lung disease. Int J Nurs Sci. 2019;6:38–42. 10.1016/j.ijnss.2018.11.005. 31406867 PMC6608652

[18] Xie Y, Zhang P, Ren J, et al. Patient-reported outcome scale for idiopathic pulmonary fibrosis: development and validation in China. J Evid Based Med. 2024;17:758–70. 10.1111/jebm.12659. 39708363

[19] Kirsten D, de Vries U, Costabel U, et al. A new tool to assess quality of life in patients with idiopathic pulmonary fibrosis or non-specific interstitial pneumonia. Pneumologie. 2022;76:25–34. 10.1055/a-1579-7618. 34521147 PMC8789482

[20] Prior TS, Hoyer N, Shaker SB, et al. Validation of the IPF-specific version of St. George’s Respiratory Questionnaire. Respir Res. 2019;20:199. 10.1186/s12931-019-1169-9. 31462235 PMC6714302

[21] Prior TS, Hoyer N, Shaker SB, et al. Validation of a derived version of the IPF-specific Saint George’s Respiratory Questionnaire. Respir Res. 2021;22:259. 10.1186/s12931-021-01853-2. 34610840 PMC8491388

[22] Yorke J, Jones PW, Swigris JJ. Development and validity testing of an IPF-specific version of the St George’s Respiratory Questionnaire. Thorax. 2010;65:921–6. 10.1136/thx.2010.139121. 20861296 PMC3697105

[23] Pan RL.Validation of the Chinese version of ATAQ-IPF in Chinese patients with Interstitial Lung Disease. Dissertation, Peking Union Medical College, 2014.

[24] Ningzi Z, Lijian P, Pin L, et al. Establishment and evaluation of IPF-TCM-HRQOL32: A health-related quality of life of TCM scale for patients with idiopathic pulmonary fibrosis[J]. Chin J Trad Chin Med. 2016;31(05):1638–42.

[25] Yiting C, Jian P, Lanjun S, Yan H, Guixing Y, Zheng L, et al. Introduction of COSMIN method: interpretation of COSMIN method combined with a systematic evaluation example[J]. Chin Nurs Res. 2021;35(9):1505–10. 10.12102/j.issn.1009-6493.2021.09.001.

[26] Jian Peng, Lanjun Shen, Yiting Chen, et al. An overview of the COSMIN-RoB checklist and the interpretation of it in evaluating therisk of bias of studies on internal structure. Chinese J Evid Based Med. 2020;20(10):1234–40. 10.7507/1672-2531.202003163.

[27] Jian Peng, Lanjun Shen, Yiting Chen, et al. Interpretation of COSMIN risk of bias checklist in evaluating risk of bias of studies onreliability, measurement error and criteria validity of patient-reported outcomemeasures. Chin J Evid Based Med. 2020;20(11):1340–4. 10.7507/1672-2531.202003164.

