# Supplementary material for: Quality of life instruments in idiopathic pulmonary fibrosis: a COSMIN-based systematic review
Source: BMC Pulm Med. 2026 Jul 31;26:396. doi: 10.1186/s12890-026-04550-2 (PMC13540806; doi:10.1186/s12890-026-04550-2)
Supplement: Supplementary file 1 [file 12890_2026_4550_MOESM1_ESM.docx]

**Appendix A. Full search strategies**

****PubMed****

**((("Idiopathic Pulmonary Fibrosis"[MeSH Terms] OR "Idiopathic Pulmonary Fibrosis"[Title/Abstract] OR "Pulmonary Fibrosis, Idiopathic"[Title/Abstract] OR "Cryptogenic Fibrosing Alveolitis"[Title/Abstract] OR "Fibrosing Alveolitis, Cryptogenic"[Title/Abstract] OR "Fibrocystic Pulmonary Dysplasia"[Title/Abstract] OR "Dysplasia, Fibrocystic Pulmonary"[Title/Abstract] OR "Idiopathic Fibrosing Alveolitis, Chronic Form"[Title/Abstract] OR "Familial Idiopathic Pulmonary Fibrosis"[Title/Abstract] OR "Idiopathic Pulmonary Fibrosis, Familial"[Title/Abstract] OR "Usual Interstitial Pneumonia"[Title/Abstract] OR "Interstitial Pneumonia, Usual"[Title/Abstract] OR "Usual Interstitial Pneumonitis"[Title/Abstract] OR "Interstitial Pneumonitis, Usual"[Title/Abstract])) AND (("quality of life"[Title/Abstract] OR "QOL"[Title/Abstract])) AND (("tool"[Title/Abstract] OR "scale"[Title/Abstract] OR "instrument"[Title/Abstract] OR "questionnaire*"[Title/Abstract]))**

****Embase****

**('idiopathic pulmonary fibrosis'/exp OR 'idiopathic pulmonary fibrosis':ab,ti OR 'pulmonary fibrosis, idiopathic':ab,ti OR 'cryptogenic fibrosing alveolitis':ab,ti OR 'usual interstitial pneumonia':ab,ti) AND ('quality of life'/exp OR 'quality of life':ab,ti OR 'qol':ab,ti) AND ('tool':ab,ti OR 'scale':ab,ti OR 'instrument':ab,ti OR 'questionnaire*':ab,ti)**

****Web of Science****

**(TS=("idiopathic pulmonary fibrosis" OR "pulmonary fibrosis, idiopathic" OR "cryptogenic fibrosing alveolitis" OR "usual interstitial pneumonia")) AND (TS=("quality of life" OR "QOL")) AND (TS=("tool" OR "scale" OR "instrument" OR "questionnaire*"))**

****CINAHL****

**(MH "Idiopathic Pulmonary Fibrosis" OR TI "idiopathic pulmonary fibrosis" OR AB "idiopathic pulmonary fibrosis" OR TI "usual interstitial pneumonia" OR AB "usual interstitial pneumonia") AND (MH "Quality of Life" OR TI "quality of life" OR AB "quality of life" OR TI "QOL" OR AB "QOL") AND (TI "tool" OR AB "tool" OR TI "scale" OR AB "scale" OR TI "instrument" OR AB "instrument" OR TI "questionnaire" OR AB "questionnaire")**

****CNKI****

**(SU='idiopathic pulmonary fibrosis' OR SU='idiopathic interstitial pneumonia' OR SU='interstitial lung disease' OR SU='pulmonary fibrosis' OR TI='idiopathic pulmonary fibrosis' OR TI='idiopathic interstitial pneumonia' OR TI='interstitial lung disease' OR TI='pulmonary fibrosis' OR AB='idiopathic pulmonary fibrosis' OR AB='idiopathic interstitial pneumonia' OR AB='interstitial lung disease' OR AB='pulmonary fibrosis') AND (SU='quality of life' OR SU='health-related quality of life' OR TI='quality of life' OR TI='health-related quality of life' OR AB='quality of life' OR AB='health-related quality of life') AND (SU='questionnaire' OR SU='scale' OR SU='instrument' OR TI='questionnaire' OR TI='scale' OR TI='instrument' OR AB='questionnaire' OR AB='scale' OR AB='instrument' OR TI='reliability' OR TI='validity' OR TI='responsiveness' OR TI='internal consistency' OR AB='reliability' OR AB='validity' OR AB='responsiveness' OR AB='internal consistency')**

**(Note: Searches were conducted using Chinese translations of the above terms; field codes: SU = Subject, TI = Title, AB = Abstract.)**

****Wanfang Database****

**(Topic:"idiopathic pulmonary fibrosis" OR Topic:"idiopathic interstitial pneumonia" OR Topic:"interstitial lung disease" OR Topic:"pulmonary fibrosis") AND (Topic:"quality of life" OR Topic:"health-related quality of life") AND (Topic:"questionnaire" OR Topic:"scale" OR Topic:"instrument" OR Topic:"reliability" OR Topic:"validity" OR Topic:"responsiveness" OR Topic:"internal consistency")**

**(Note: Searches were conducted using Chinese translations of the above terms; field code: Topic.)**

****VIP Database****

**(M=idiopathic pulmonary fibrosis OR M=idiopathic interstitial pneumonia OR M=interstitial lung disease OR M=pulmonary fibrosis) AND (M=quality of life OR M=health-related quality of life) AND (M=questionnaire OR M=scale OR M=instrument OR M=reliability OR M=validity OR M=responsiveness OR M=internal consistency)**

**(Note: Searches were conducted using Chinese translations of the above terms; field code: M = Subject.)**

****Chinese Biomedical Literature Database (CBM)****

**("idiopathic pulmonary fibrosis"[Common Field] OR "idiopathic interstitial pneumonia"[Common Field] OR "interstitial lung disease"[Common Field] OR "pulmonary fibrosis"[Common Field]) AND ("quality of life"[Common Field] OR "health-related quality of life"[Common Field]) AND ("questionnaire"[Common Field] OR "scale"[Common Field] OR "instrument"[Common Field] OR "reliability"[Common Field] OR "validity"[Common Field] OR "responsiveness"[Common Field] OR "internal consistency"[Common Field])**

**(Note: Searches were conducted using Chinese translations of the above terms; field code: Common Field.)**

**Search period: From January 1, 2010 to December 28, 2025.**
